# The CARe Burn Scale—Adult Form: Identifying the Responsiveness and Minimal Important Difference (MID) Values of a Patient Reported Outcome Measure (PROM) to Assess Quality of Life for Adults with a Burn Injury

**DOI:** 10.3390/ebj3010019

**Published:** 2022-03-10

**Authors:** Catrin Griffiths, Philippa Tollow, Danielle Cox, Paul White, Timothy Pickles, Diana Harcourt

**Affiliations:** 1 Centre for Appearance Research (CAR), University of the West of England (UWE), Bristol BS16 1QY, UK; catrin.griffiths@uwe.ac.uk (C.G.); pippa.tollow@uwe.ac.uk (P.T.); 2 Office for National Statistics, Newport NP10 8XG, UK; danielle.cox@ons.gov.uk; 3 Mathematics and Statistics Research Group, Department of Computer Science and Creative Technologies, University of the West of England (UWE), Bristol BS16 1QY, UK; paul.white@uwe.ac.uk; 4 Centre for Trials Research (CTR), Cardiff University, Cardiff CF14 4YS, UK; pickleste@cardiff.ac.uk

**Keywords:** burn injuries, scars, patient-reported outcome measures, scar management

## Abstract

The CARe Burn Scales are a suite of burn-specific PROMs for adults, children, young people, and parents affected by burns. This study aimed to determine the responsiveness and minimal important difference (MID) values of the Adult Form for use in adult burn care and research. Participants were recruited by 11 UK Burn Services. They completed online or paper versions of the CARe Burn Scale –Adult Form and a set of appropriate comparison validated measures and anchor questions at baseline (T1, up to 4 weeks post-burn), 3 months (T2), and 6 months post-burn (T3). A total of 269 participants took part at baseline and 226 (84%) were retained at the 6-month follow-up. Spearman’s correlation analysis and effect sizes based on Cohen’s d thresholds were reported and MID values calculated. MID values were created for all subscales and ranged from 4–15. The CARe Burn Scale–Adult Form is responsive to change over time and can therefore be used to reliably inform the management of adults’ burn injury treatment and recovery. It is freely available for clinical and research use.

## 1. Introduction

The impact of a burn injury can be extensive and enduring. The injury, its treatment, and subsequent scarring can be associated with physical symptoms including pain, sensitivity, itching, and restricted mobility, as well as psycho-social difficulties such as trauma symptoms, social anxiety, and sleep disturbance [1,2,3,4]. Unwanted reactions, comments, and unsolicited questions from other people can lead to social avoidance, withdrawal, fear of being negatively judged, and detrimental impacts on self-esteem and quality of life [5]. Scars and an altered appearance can also impact body image and, for some, present difficulties around work and concerns around establishing and maintaining romantic and intimate relationships [2]. Whilst some adults manage the challenges they face very well and may demonstrate positive outcomes and personal growth [6,7], others struggle to make the adjustment and redefine a sense of normality [8].

Given the potentially complex and wide-ranging consequences of burn injuries and scarring, it is essential that health professionals and researchers can easily and effectively assess patients’ wellbeing and adjustment in order to appropriately manage any support needs and reduce the likelihood and impact of long-term difficulties. Patient reported outcome measures (PROMs) can identify patients’ needs and be used to assess the impact of interventions in both clinical and research settings.

Effective and reliable PROMs must incorporate the issues that are important to the patients themselves [9]. Condition-specific PROMS are tailored to the experiences of a specific patient group (such as those affected by a burn) and therefore likely to be sensitive to change [10]. Using a combination of condition-specific and generic PROMs can assess issues that are unique to a particular patient group whilst also capturing more universal outcomes that enable comparisons with other groups.

The UK Department of Health [11] recommended that PROMs should be used to evaluate outcomes and inform healthcare evaluation, commissioning, and regulatory decision making. However, they have not been routinely used in UK burn care [12], and the need for new PROMs to enable rigorous measurement was reinforced by the UK National Burn Care Standards [13]. We have therefore conducted a programme of work to develop and validate a portfolio of burn-specific age-appropriate PROMs, known as the CARe Burn Scales (Adult, Child, Young Person, and Parent Forms) [14,15,16]. These were created in accordance with a recognised, rigorous development and validation process [17] based on guidelines for the development of health outcome measures [18]. This involved item generation through literature reviews, interviews with patients and health professionals, and a rigorous process of psychometric testing (using Rasch analysis) (see 14, 15 for further details).

The CARe Burn Scale—Adult Form has 59 items across 14 domains of quality of life: 12 individual scales (Wound/Scar Discomfort, Wound/Scar Dissatisfaction, Physical Wellbeing, Social Situations, Self-Worth, Negative Mood, Work Life, Family Support, Friend Support, Intimacy, Trauma Symptoms, and Positive Growth) and 2 checklists (Wound/Scar Treatment and Avoidance Behaviours). Checklists are not psychometrically valid, so they are used for information rather than measurement. Uniquely, the CARe Burn Scale is suitable for use from the time of injury and throughout the patient’s recovery and beyond since it includes both the wound and scar stages of the burn injury. This makes it a potentially valuable asset for clinicians looking to assess, monitor, and manage the ongoing impact of a burn and subsequent scarring.

A study with 304 adults with burn injuries demonstrated the construct reliability, internal consistency reliability, and validity of the CARe Burn Scale—Adult Form {see 14 for details}. However, further testing is needed to assess the scale’s responsiveness (i.e., its ability to validly detect a change in patient reported outcomes over time) and determine their minimal important differences (MIDs) in order to establish their clinical efficacy and value in longitudinal research [19]. The MID is the smallest identifiable change score on a domain that patients perceive as being meaningfully important to them [20]. We have previously reported the responsiveness and MID values for the CARe Burn Scales for children, young people, and parents (see 15).

## 2. Materials and Methods

All necessary University and NHS ethics approvals were granted (NHS REC reference: 15/SW/0263). Participants provided written or online informed consent, depending on whether they completed paper-based or online versions of the questionnaires.

Recruitment took place through 11 NHS Burn services across England, Wales, and Scotland. A longitudinal design was used, with data collection at baseline (T1, up to 4 weeks post-burn), 3 months (T2), and 6 months post-burn (T3). Following the COSMIN checklist for the design of responsiveness studies [21], the CARe Burn Scale—Adult Form was tested in comparison to other validated measures which assess similar constructs to determine evidence of responsiveness. Further analysis identified the minimal important difference (MID) values of each subscale within the CARe Burn Scale—Adult Form.

### 2.1. Eligibility Criteria

Participants were adults aged 18 years old or over and treated by the recruiting NHS Burn Service for a burn injury of any size or location on the body that was sustained up to 4 weeks earlier. Participants needed a sufficient comprehension of English to complete the questionnaires.

### 2.2. Measures

The baseline questionnaire (T1) collected demographic information including the participant’s age, gender, ethnicity, education, time since burn, cause of burn, and treatments received. At each time point, the questionnaire pack included the CARe Burn Scale—Adult Form and the relevant comparison measures (see Table 1).

Since the purpose of this study was to identify the responsiveness of the CARe Burn Scale—Adult Form, only the 12 individual subscales were analysed (not the two checklists). The 12 subscales are scored from 0–100, with higher scores reflecting better outcomes (scoring instructions can be accessed via www.careburnscales.org.uk, accessed on 9 March 2022). They were compared with outcome measures (see below and in Table 1) chosen through a team collective decision-making process on the basis of their psychometric properties and subject domain knowledge, which also indicated the expected direction of correlation.

The EQ-5D-5L [22], consisting of the EQ-5D descriptive system (a 5-item measure of impairments in body function with five subscales (mobility, self-care, usual activities, pain/discomfort, and anxiety/depression), each measured on a 5-point Likert scale ranging from 1 (No problems) to 5 (Extreme problems) with a higher score indicating worse outcomes)), a summary index score of the five items with higher scores reflecting a better quality of life, and the EQ Visual Analogue Scale (EQ VAS—a 1-item measure of patient self-rated health on a scale from 0 (The worst health you can imagine) to 100 (The best health you can imagine)). It has shown good reliability in adults [23], validity in adult burn patients [24], and responsiveness in adult stroke patients [25].

The Burn Specific Health Scale-Brief (BSHS-B) [26], a 40-item measure of the quality of life after a burn injury with nine subscales (Heat Sensitivity, Affect, Hand Function, Treatment Regimens, Work, Sexuality, Interpersonal Relationships, Simple Abilities, and Body Image), with items measured on a 5-point Likert scale ranging from 0 (Extremely) to 4 (None at all) with a higher score indicating better outcomes. In studies with adult burn patients, it has shown good reliability, validity [27], and responsiveness [28].

The Multidimensional Scale of Perceived Social Support (MSPSS) [29], a 12-item measure of social support with three subscales (Family, Friends, and Significant Other), with items measured on a 7-point Likert scale ranging from 1 (Very strongly disagree) to 7 (Very strongly agree) with a higher score indicating better outcomes. It has shown good reliability and validity in adults [29].

The Mental Health Inventory (MHI) [30]—the Depression (four items), Anxiety (three items), and Behavioural Control (four items) subscales were used as a measure of psychological distress and wellbeing. These consist of 11-items measured on a 6-point Likert scale ranging from 1 (All of the time) to 6 (None of the time) with higher scores indicating better outcomes. It has shown good reliability and validity in adults [31].

The PTSD Checklist Civilian Version (PCL-C) [32], a 17-item measure of symptoms of Post-Traumatic Stress Disorder, with items measured on a 5-point Likert scale ranging from 1 (Not at all) to 5 (Extremely) with a higher score indicating poorer outcomes. It has shown good reliability in adults [33] and validity in adult burn patients [34].

The Post-traumatic Growth Inventory-Short Form (PTGI-SF) [35], a 10-item measure of post-traumatic growth with items presented on a 6-point Likert scale ranging from 0 (I did not experience this change) to 5 (I experienced this change to a very great degree). Higher scores indicate better outcomes, and it has shown good reliability and validity in adult burn patients [36].

### 2.3. Anchor Questions to Calculate Minimal Important Difference (MID) Values

For the anchor-based MID analysis, a single item transition question for each subscale was included at T2 and T3. These asked whether the participant thought they had changed in the domain being assessed by that subscale (e.g., Since the last time you did this survey, how much has your physical health changed?). Each transition question had five response categories (a little better, a lot better, no change, a little worse, and a lot worse), with the exact wording of the question adjusted to suit each particular domain.

### 2.4. Procedure

We sought to recruit consecutive patients with burn injuries that ranged in size (TBSA) and location. Participants were informed that the study was testing a questionnaire that measured the health and well-being of adults living with a burn injury. Participants chose whether to complete the questionnaire on paper or online via a link to a secure online platform (www.qualtrics.com, accessed on 1 November 2018).

Paper questionnaires were handed out by burn health professionals to eligible participants in outpatient clinics and posted to those who had been identified through patient database searches, with an option to use a web survey link to complete the questionnaire online if they preferred. Some sites also displayed study posters in their outpatient clinics, promoting the link to the online survey.

At both follow-up points (T2 and T3), participants were sent either a paper questionnaire pack to complete and return using a pre-paid envelope or a web link to complete the questionnaire online, depending on their preference expressed at T1. Those who had not completed their follow-up questionnaire within one week were reminded via email, telephone call, or post. Participants received a £10 online shopping voucher for taking part at each time point (T1, T2, and T3).

### 2.5. Statistical Analysis

Sample size: This study is predicated on an assumption of a mutually correlated system between a burn scale, its comparator, changes in the burn scale, changes in the comparator, and the single item anchor measure. In a two-sided test of correlation, sample sizes of 84, 96, 112, and 138 would have at least 80, 85, 90, and 95% power, respectively, for a correlation of at least 0.3 (alpha = 0.05).

Responsiveness analysis: Three change scores were calculated for each of the CARe Burn Scale—Adult Form subscales and the related comparison measures by subtracting the participant’s subscale scores from one another at each time point (i.e., T3–T2, T2–T1, and T3-T1). All the subscales and comparison measures were computed in accordance with the scoring instructions. Spearman’s correlations were conducted between the change scores for the CARe Burn Scale—Adult Form subscales and the comparison measures, and related constructs were compared for each change score time point [37]. Analyses were undertaken using IBM SPSS Statistics [38].

Cohen’s criteria were used as a guide for the magnitude of correlations. Absolute values of a correlation between 0.1 and 0.3 are viewed as being “small”, with values between 0.3 and 0.5 considered “medium”, and values above 0.5 as being “large” [39].

Missing data: Little’s MCAR test was used to examine the data and the pattern of missing values at T2 and T3 for each subscale in relation to the baseline. This test result was consistent with the data missing being completely at random (*p* > 0.05). On this basis, the data was analysed on an all-available case basis, maximising the amount of data in any analysis.

### 2.6. Hypotheses

As per the COSMIN guidelines, responsiveness is concerned with the size and direction of the correlations between changes in the construct and changes in the comparison measure [21]. In these respects, at least moderate correlations (approximately 0.3) would be expected. Whether the correlations statistically differ from zero, although important, is of lesser concern when providing evidence of responsiveness [21].

Hypotheses were determined based on the premise that constructs in the CARe Burn Scale—Adult Form would moderately correlate with similar constructs in other validated PROMs. The expected direction of effects was determined a priori, but they were not published in a publicly available protocol prior to the study’s end.

### 2.7. Specifically

The hypotheses related to the change scores for each subscale of the CARe Burn Scale—Adult Form were:

- Wound/Scar Discomfort would have moderate and negative correlations with the EQ-5D-5L Pain Discomfort subscale.
- Wound/Scar Dissatisfaction would have moderate and positive correlations with the Burn Specific Health Scale Brief Body Image subscale.
- Physical Well-being would have moderate and positive correlations with the EQ-5D-5L—Summary Index.
- The Social Situations scale reflects how confident patients are with their scar/body image in social settings and it was therefore hypothesised that it would have moderate and positive correlations with the Burn Specific Health Scale Brief Body Image subscale.
- Self-Worth would have moderate and positive correlations with the Mental Health Inventory Depression subscale.
- Negative Mood would have moderate and positive correlations with the Mental Health Inventory Depression subscale.
- Work Life would have moderate and positive correlations with the Burn Specific Health Scale Brief Interpersonal Relationships subscale.
- Family would have moderate and positive correlations with the Multidimensional Scale of Perceived Social Support Family subscale.
- Friendship Support would have moderate and positive correlations with the Multidimensional Scale of Perceived Social Support Friend subscale.
- The Intimacy scale reflects how confident respondents are with their scar/body image in intimate situations. It was therefore hypothesised to have moderate and positive correlations with the Burn Specific Health Scale Brief Body Image subscale.
- Trauma Symptoms would have moderate and negative correlations with the PTSD Checklist Civilian version.
- Positive Growth would have moderate and positive correlations with the Post-traumatic Growth Inventory-Short Form.

### 2.8. MID Analysis

There are various methods for calculating the MID. Anchor-based methods involve asking patients an anchor question where they report the degree to which their health has changed. Alternatively, distribution-based MID calculations are based on the statistical attributes of the data (i.e., means and standard deviations). In this study, MIDs were derived from both anchor-based and distribution-based methods, and these results were then triangulated to determine final MID values, as recommended by Revicki et al. [20]. Details of the methods used to calculate the MIDS using these approaches are in the Appendix A.

## 3. Results

Participant demographics are presented in Table 2. There were 269 participants at baseline (T1), 230 (85% retained) at T2, and 226 (84% retained) at T3.

### 3.1. Responsiveness Analysis

Table 3 provides results of the means and standard deviations, Cronbach’s alphas, and level of missing data for each subscale of the Adult Form. A threshold of >0.7 was used to indicate acceptable values for Cronbach’s alpha.

All subscales exceeded the criteria for validity and reliability. Scale reliability was supported by high Cronbach’s alpha coefficients (*p* > 0.80 for all except Wound/Scar Discomfort at T1). The level of missing data was good for all subscales at T1, except for Work Life (52.0%) and Intimacy (24.7%). Not all participants were economically active since some were unemployed, some were retired, and some did not return to work straight after a burn. This likely explains the level of missing data for the Work Life subscale. Similarly, not all patients were in a relationship and others may not wish to discuss their intimate life, so the degree of missing data for the Intimacy subscale is not surprising. Missing data increased at follow-up time points for most subscales, ranging from 16.4% (Negative Mood and Wound/Scar Dissatisfaction at T2) to 23.3% (Family Support at T3), which is in line with the small participant attrition over the follow-up period.

All but one of the subscale scores improved over each time point, reflecting better health outcomes. The exception was Family Support which showed a slight reduction for the mean at T3 (83.99) compared to the mean at T1 (baseline: 85.73). Correlations between the CARe Burn Scale—Adult Form and the comparison measure at each time point are shown in Table 4.

Scale responsiveness was supported by the correlations between the change scores of the CARe Burn Scale—Adult Form subscales and the other validated quality of life measures (see Table 5).

As predicted, Wound/Scar Discomfort had moderate negative correlations with the EQ-5D-5L Pain Discomfort subscale, and Wound/Scar Dissatisfaction had low to moderate positive correlations with the Burn Specific Health Scale Brief (BSHS-B) Body Image subscale. Physical well-being had moderate positive correlations with the EQ-5D-5L Summary Index. Social situations had low positive correlations with the Burn Specific Health Scale Brief Body Image subscale. Self-Worth and Negative Mood had moderate positive correlations with the Mental Health Inventory Depression subscale. Work Life had low to moderate positive correlations with the Burn Specific Health Scale Brief Interpersonal Relationships subscale. Family Support had low to moderate positive correlations with the Multidimensional Scale of Perceived Social Support Family subscale. Friendships had low positive correlations with the Multidimensional Scale of Perceived Social Support Friend subscale. Intimacy had moderate and positive correlations with the Burn Specific Health Scale Brief Body Image subscale. Trauma Symptoms had moderate to strong negative correlations with the PTSD Checklist Civilian Version and Positive Growth had low to moderate positive correlations with the Post-traumatic Growth Inventory Short Form. Social Situations and Friend Support were the only subscales not to obtain any moderate correlations.

### 3.2. MID Analysis

Anchor-based approach: Adult Form correlations with anchor questions at each time point are shown in the Appendix A, Table A1. As expected, correlations between the anchor and its related domain change score were negative and were low to moderate, ranging between 0.1 and 0.4 (see Appendix A Table A2).

All MID values derived from the T2 anchor question produced similar levels of accuracy at T3 in distinguishing between ‘no change’, ‘small change’, and ‘large change’, providing validation of the MID values (see Appendix A Table A3) which ranged from 4 (Trauma Symptoms) to 15 (Work Life). Overall accuracy ranged from 49% to 76%, with an average of 60%. The percentage of participants reporting a small change ranged from 25% (Work Life) to 66% (Friend Support), with an average of 46%.

Regarding the distribution-based approach, the overall accuracy ranged from 39% to 69% (average 55%) (see Appendix A Table A4). The percentage of participants reporting a small change ranged from 22% to 79%, with an average of 41% across the subscales.

All of the MID values derived using the distribution-based method were identical to those using the anchor-based method, except for Work Life at T3 where the distribution-based method calculated a MID of 15, compared to 12 from the anchor-based method. Anchor-based MIDs were retained for the final set of MID values since, as stated earlier, anchor-based MIDs are based on the self-reported change in the domain (see Table 6 for final MID values).

## 4. Discussion

Overall, this study provides evidence for the responsiveness of the CARe Burn Scale—Adult Form to identify changes in outcomes amongst adult burn patients over the first 6 months following injury. The majority (10 out of 12) of the subscales had at least one or more moderate change score correlations with the prior reasoned comparator quality of life measure. The correlations with the comparison measures (reported in Table 4) were in the hypothesised direction, but there was variation in their strength. Some were moderate to high, but others had small correlations. This is a limitation.

The CARe Burn Scale—Adult Form was developed and validated with adult burn patients who had received treatment in the UK’s NHS Burn Service. They played a vital part in the creation of the PROM, informing item generation and reviewing and commenting on draft versions of the scale. The CARe Burn Scale—Adult Form, therefore, measures a broad range of quality-of-life domains that reflect key experiences that are pertinent to adults with a burn injury. Importantly, they highlighted the need to include both the wound and scar stages of injury recovery and trauma symptoms, and to ensure the PROM could recognise positive outcomes and growth such as increased confidence, greater empathy towards those that look different, and greater appreciation for life. The CARe Burn Scale—Adult Form is the first burn-specific PROM for adults to refer to both the wound and scar stage of recovery. This makes it particularly useful for assessing the impact of a burn injury over time from the initial injury, throughout the recovery period, and beyond. It is also novel in including a specific sub-scale to measure positive growth. Post-traumatic growth is an important topic for burns research [6] and including this within a PROM used routinely in care could facilitate attention being given to this often-overlooked area.

Some of the domains included in the CARe Burn Scale—Adult Form are similar to those in existing burn-specific PROMs such as Wound/Scar Dissatisfaction [26,40,41,42,43], Physical Abilities [26,40,41,42,43], Wound/Scar Discomfort [41,42,43], Confidence in Social Situations [41,42,43,44], Friendships [26,40,41,42,43,44], Family [26,40,41,42,43,44], Work [26,41,42,43,44], Intimacy [26,40,41,42,43,44], and Negative Mood [26,40,41,42,43,44].

However, since the CARe Burn Scales were developed using in-depth interviews with patients and health professionals to inform the conceptual framework and PROM items, rather than relying on existing PROMs or conceptual frameworks, this method led to additional new domains which are not included in other existing scales such as the Abbreviated Burn Specific Health Scale (BSHS-A) [40], the Burn Specific Health Scale—Brief (BSHS-B) [26], the Young Adult Burn Outcome Questionnaire (YABOQ) [43], the Adult Burn Outcome Questionnaire (YABOQ) Short Form [41], the Coping with Burns Questionnaire [45], the Life Impact Burn Recovery Evaluation (LIBRE) [44], and the Brisbane Burn Scar Impact Profile [42]. The domains which are unique to the CARe Burn Scale—Adult Form are Positive Growth (i.e., life being more meaningful or feeling a better person after a burn injury), Self-Worth (i.e., feeling confident, happy), Trauma Symptoms (i.e., feeling upset, short tempered, experiencing bad dreams, or flashbacks/vivid memories), Avoidance Behaviours (i.e., avoiding looking at or touching burn wounds/scars, covering up wounds/scars or avoiding certain social activities because of their wounds/scars), and Wound/Scar Treatments (i.e., whether treatments such as dressing changes, creaming/massage, and physiotherapy exercises bother the patient). The Brisbane Scar Impact Profile [46] does include an item on scar treatment (pressure garments, exercises, and creams). The identification of new domains during the development of the CARe Burn Scale—Adult Form reinforces the importance of using in-depth interviews when creating PROMs. This ensures that the scale includes the range of health outcomes reported by patients as key to their health. A further advantage of the CARe Burn Scale—Adult Form is that it is freely available for download (via www.careburnscales.org.uk (accessed on 3 March 2022)) and users are able to score the data themselves using the scoring sheets downloadable from the same website.

Importantly, calculating MID values for PROMs is still innovative in psychometrics and our study is one of few psychometric studies with burn populations to include MID values. We hope that MID values for other scales used in burns research will be available in the future. These are key when using PROMs to effectively identify patient progress and treatment effectiveness. They are therefore extremely useful for clinicians since they can help to indicate whether an individual has made a meaningful change on any particular subscale, whether a change in their management is warranted, and/or whether a particular treatment is having an effect, and thereby inform evidence-based decision making. The majority of MID values correctly identified 25–66% of participants who reported a small improvement, but this means that around 34–75% were not identified correctly. The MID values were developed using T2 data and subjected to validation using data at T3. Generally, the predictive accuracy of the MIDs at T3 is not overly discrepant from the accuracy at T2 for the single anchors providing validation on predictive accuracy. The distributional approach to identify an appropriate MID for each subscale was employed using the T1 and T2 data. These analyses triangulated the findings from the anchor approach. The MIDS were further validated using the data from T3. In summary, the two approaches coupled with the validation gave community agreement on the MID for the subscales. It is noted that at T3, the percentage correct in the ‘No Change Category’ for the anchor method is always greater than or equal to the percentage correct in the small change category. This shows that the MID thresholds have not been set too low, i.e., not claiming too many to have changed when they in fact report no change, providing extra confidence that safeguards against false findings for researchers and clinicians.

It is important to note that, like all scales of measurement, the MID is context-dependent and MID values are likely to vary depending on patient demographics, baseline data, and the anchors used [47]. The MID values we identified may therefore have been different if alternative anchors and a different population were involved. Furthermore, the time post-burn could be relevant since a large change over a longer period of time might be considered indicative of a greater benefit than a similar change over a short period of time. When interpreting the current findings, it is important to consider these points and the limitations of our sample (detailed below) which may not be representative of adult burn populations in the UK. Furthermore, other factors that were not assessed in this study could impact MID. For example, the size (Total Burn Surface Area: TBSA) or cause of the burn, treatment received, indicators of deprivation, and psychosocial factors including coping strategies were not considered in this analysis.

### 4.1. Strengths

This study has a number of strengths, including the high participant retention rates at each follow-up and the recruitment of adults treated by burn services across England, Scotland, and Wales, rather than relying on recruitment from a single burn service or a limited geographical region. Participants were given a choice of completing a paper-based or online set of measures. The majority completed an electronic version of the measures at T1 (53.1%), T2 (57.8%), and T3 (59.6%), with an increasing number of respondents choosing to complete the electronic version (rather than a paper questionnaire) at each time point. This indicates the benefits of offering participants a choice about how to take part and suggests that, when using the CARe Burns Scale with adults in future research and clinical work, patients may be likely to find online completion acceptable.

### 4.2. Limitations

The current study included data collection up to six months post-burn. Future research could examine the ability of the CARe Burn Scale—Adult Form to identify clinical changes over a longer period of time to ascertain the long-term impact of a burn.

Despite recruiting through burn services across England, Scotland, and Wales, the proportion of participants reporting their ethnicity as being other than White British was very low. Further evidence is needed of the scale’s use with a larger sample of adults from ethnic minority backgrounds. Furthermore, we do not have data from each site regarding the proportion of the total population sampled, the number of study packs given out, or how many patients would have been eligible. This means that, although the intention was for consecutive eligible patients at each site to be invited to participate (in order to minimise selection bias), we cannot be sure that the participants are representative of those treated at each site or by burn services nationally.

Participants were asked to report their TBSA but there was a considerable amount of missing data, which is a limitation of the study and has precluded the inclusion of TBSA in the analysis (see above). Anecdotal evidence suggests that patients are often unaware of the TBSA of their injury or report it inaccurately. Gathering this data from patient records would be advantageous in future research but was not possible in the current study.

Finally, further testing is warranted if the scale is to be used elsewhere. Translation studies are needed if it is to be used with non-English speaking patients, and its value as a tool that can assess patient-reported outcomes in different cultures needs to be explored. To date, it has been translated and validated in Finnish [48].

### 4.3. Using the Adult Form in Clinical Practice and Research

The full set of CARe Burn Scales and scoring spreadsheets are freely available at www.careburnscales.org.uk (accessed on 3 March 2022). They are intended to be used to identify adult burn patients’ clinical and support needs and ascertain therapeutic progress and conduct service evaluation and research. Clinicians and researchers can use the MID values to identify whether the quality of life of the adult patients they are working with has meaningfully changed between two time points. In order to use the MID values reported in this study, we encourage health professionals to use the scoring templates available at www.careburnscales.org.uk (accessed on 3 March 2022) to establish a score for each subscale and then compare these scores with the relevant MID values reported in this paper. If the absolute difference between the two time periods is greater than or equal to the MID value, it can be ascertained that that person has meaningfully changed (improved/deteriorated depending on whether scores have increased or decreased in the follow-up time point) on that subscale.

The potential value of using the CARe Burn Scale—Adult Form to inform shared treatment decision making is worthy of investigation.

## 5. Conclusions

The CARe Burn Scale—Adult Form is responsive and can detect changes over time. It is now freely available as part of a set of burn-specific PROMs for use in clinical settings and research to identify patients’ needs and therapeutic progress, conduct service evaluation, and compare outcomes at different burn centres (see www.careburnscales.org.uk (accessed on 3 March 2022) to obtain the full set of CARe Burn Scales).

## Figures and Tables

**Table 1:** The CARe Burn Scale—Adult Form subscales and their comparison outcome measures.

CARe Burn Scale—Adult Form Subscale	Comparison Outcome Measure
Wound/Scar Discomfort	EQ-5D-5L (Pain Discomfort subscale)
Wound/Scar Dissatisfaction	Burn Specific Health Scale Brief (Body Image subscale)
Physical Well-being	EQ-5D-5L (Summary Index)
Social Situations	Burn Specific Health Scale Brief (Body Image subscale)
Self-Worth	Mental Health Inventory (Depression subscale)
Negative Mood	Mental Health Inventory (Depression subscale)
Work Life	Burn Specific Health Scale Brief (Interpersonal Relationships subscale)
Family Support	Multidimensional Scale of Perceived Social Support (Family subscale)
Friend Support	Multidimensional Scale of Perceived Social Support (Friend subscale)
Intimacy	Burn Specific Health Scale Brief (Body Image subscale)
Trauma Symptoms	PTSD Checklist Civilian Version
Positive Growth	Post-traumatic Growth Inventory-Short Form

**Table 2:** Demographic information of participants completing the CARe Burn Scale—Adult Form at baseline (T1).

		N	%
Age	Mean 44.15 (SD 27.33), range 18 to 84	269	100
Gender	Male	137	49.8
	Female	125	45.5
Relationship Status	Married	114	41.5
	Civil Partnership	5	1.8
	Single, never married	72	26.2
	Separated	6	2.2
	Divorced	9	3.3
	Cohabiting	36	13.1
	In a relationship but not living together	16	5.8
	Widow/Widower	9	3.3
Ethnicity	White British	233	84.7
	White Other	15	5.5
	Asian or Asian British: Indian	6	2.2
	Asian or Asian British: Bangladeshi	1	0.4
	Asian or Asian British: Other	1	0.4
	Black or Black British: Black African	3	1.1
	Black or Black British: Caribbean	1	0.4
	Mixed: White and Black African	1	0.4
	Mixed: White and Asian	1	0.4
	Mixed: Other	1	0.4
	Other	1	0.4
	Rather not say	3	1.1
Highest Level of Education	GCSEs/O-levels	74	26.9
	AS/A-levels	42	15.3
	Apprenticeship	23	8.4
	Undergraduate degree/certificate/diploma of higher education	86	31.3
	Master’s degree	26	9.5
	Doctorate/PhD	3	1.1
Time Since Injury (Days)	Mean 17.34 (SD 11.01), range 1 to 55	269	
Injury Status	Burn wound	141	51.3
	Burn scar	28	10.2
	Both wound and scar	94	34.2
	No wound or scar	4	1.5
Body Part Affected	Head or face	39	14.2
	Neck	16	5.8
	Chest	16	5.8
	Abdomen	22	8.0
	Back	16	5.8
	Lower arms	72	26.2
	Upper arms	27	9.8
	Hands	76	27.6
	Fingers	53	19.3
	Bottom	16	5.8
	Genitalia	6	2.2
	Upper legs	55	20.0
	Lower legs	59	21.5
	Feet	51	18.5
	Other	7	2.5
Cause of burn	Flame	42	15.3
	Scald/hot liquid	132	48.0
	Contact	39	14.2
	Electricity	7	2.5
	Chemical/acid	26	9.5
	Other	49	17.8
Treatments received from burns service	Surgery	46	16.7
	Physiotherapy/occupational therapy	69	25.1
	Nursing support	250	90.9
	Psychological support from a psychologist or counsellor	17	6.2
	Other support	11	4.0
Overnight hospital stay(s) (Days)	Yes (Mean 4.12 (SD 10.39), range 1–13)	73	26.5
	No	194	70.5
Surgery for burn (Number of operations)	Yes (Mean 1.12 (SD 0.42), range 1–2)	46	16.7
	No	221	80.4

NB. Percentages for “Body part affected” and “Treatments received” exceed 100% due to multiple response outcomes. For all other demographics, the percentages are the share of a given group in the whole sample of 269 participants and may not sum 100% due to missing data.

**Table 3:** Reliability and validity for the CARe Burn Scale—Adult Form at Time 1 (T1: baseline, up to 4 weeks post-burn), Time 2 (T2: 3 months post-burn), and Time 3 (T3: 6 months post-burn).

	Data Quality		Scaling Assumptions			
Subscale	N	Missing Data (%)	Possible Range	Actual Range	Mean Score (SD)	Cronbach’s Alpha
Wound/Scar Discomfort						
T1	254	7.6	0, 100	0, 100	52.19 (20.12)	0.72
T2	220	20.0	0, 100	0, 100	72.10 (22.88)	0.87
T3	220	20.0	0, 100	0, 100	77.76 (21.19)	0.87
T2–T1 change score	202	26.5	−100, 100	−53, 88	20.61 (22.83)	
T3–T2 change score	202	26.5	−100, 100	−31, 64	5.18 (14.97)	
T3–T1 change score	207	24.7	−100, 100	−53, 88	25.60 (22.09)	
Wound/Scar Dissatisfaction						
T1	263	4.4	0, 100	0, 100	61.03 (30.35)	0.89
T2	230	16.4	0, 100	0, 100	70.93 (23.80)	0.84
T3	225	18.2	0, 100	0, 100	75.41 (23.70)	0.86
T2–T1 change score	218	20.7	−100, 100	−74, 100	10.31 (24.98)	
T3–T2 change score	214	22.2	−100, 100	−66, 66	4.89 (19.41)	
T3–T1 change score	213	22.5	−100, 100	−75, 100	18.48 (27.04)	
Physical Well-being						
T1	267	2.9	0, 100	0, 100	50.87 (30.09)	0.84
T2	229	16.7	0, 100	0, 100	76.83 (27.94)	0.90
T3	226	17.8	0, 100	0, 100	78.93 (29.11)	0.93
T2–T1 change score	222	19.3	−100, 100	−100, 100	26.64 (35.95)	
T3–T2 change score	214	22.2	−100, 100	−100, 100	1.57 (29.72)	
T3–T1 change score	219	20.4	−100, 100	−100, 100	28.50 (38.45)	
Social Situations						
T1	250	9.1	0, 100	0, 100	52.91 (29.02)	0.83
T2	228	17.1	0, 100	0, 100	62.10 (32.02)	0.88
T3	223	18.9	0, 100	0, 100	67.72 (31.63)	0.89
T2–T1 change score	206	25.1	−100, 100	−100, 100	9.70 (40.50)	
T3–T2 change score	210	23.6	−100, 100	−100, 100	5.17 (24.97)	
T3–T1 change score	201	26.9	−100, 100	−100, 100	13.45 (31.68)	
Self-Worth						
T1	267	2.9	0, 100	0, 100	63.58 (27.24)	0.90
T2	228	17.1	0, 100	0, 100	66.43 (26.93)	0.90
T3	225	18.2	0, 100	0, 100	69.92 (27.51)	0.92
T2–T1 change score	220	20.0	−100, 100	−67, 93	3.27 (22.43)	
T3–T2 change score	212	22.9	−100, 100	−60, 67	3.80 (20.79)	
T3–T1 change score	217	21.1	−100, 100	−64, 100	6.77 (23.19)	
Negative Mood						
T1	269	2.2	0, 100	0, 100	75.09 (20.23)	0.82
T2	230	16.4	0, 100	22, 100	80.29 (19.70)	0.81
T3	225	18.2	0, 100	22, 100	82.60 (18.75)	0.82
T2 –T1 change score	224	18.5	−100, 100	−41, 47	5.54 (14.29)	
T3–T2 change score	214	22.2	−100, 100	−41, 48	2.22 (14.40)	
T3–T1 change score	219	20.4	−100, 100	−45, 59	8.21 (17.85)	
Work Life						
T1	132	52.0	0, 100	0, 100	82.09 (25.45)	0.87
T2	146	46.9	0, 100	0, 100	82.17 (26.98)	0.92
T3	146	46.9	0, 100	0, 100	82.49 (24.05)	0.87
T2–T1 change score	100	63.6	−100, 100	−100, 100	0.15 (28.96)	
T3–T2 change score	123	55.3	−100, 100	−54, 100	1.42 (22.60)	
T3–T1 change score	94	65.8	−100, 100	−100, 100	−1.76 (28.23)	
Family Support						
T1	261	5.1	0, 100	0, 100	85.73 (21.62)	0.88
T2	221	19.6	0, 100	0, 100	83.51 (24.39)	0.92
T3	211	23.3	0, 100	17, 100	83.99 (24.08)	0.92
T2–T1 change score	211	23.3	−100, 100	−68, 100	−2.64 (22.49)	
T3–T2 change score	195	29.1	−100, 100	−75, 62	0.10 (17.39)	
T3–T1 change score	201	26.9	−100, 100	−61, 100	−2.11 (19.20)	
Friend Support						
T1	261	6.1	0, 100	0, 100	72.74 (25.93)	0.87
T2	229	16.7	0, 100	0, 100	72.36 (25.44)	0.87
T3	224	18.5	0, 100	0, 100	74.11 (24.27)	0.87
T2–T1 change score	216	21.5	−100, 100	−100, 100	0.69 (24.92)	
T3–T2 change score	212	22.9	−100, 100	−61, 50	1.08 (19.62)	
T3–T1 change score	212	22.9	−100, 100	−100, 100	2.16 (25.32)	
Intimacy						
T1	207	24.7	0, 100	0, 100	54.64 (28.71)	0.87
T2	184	33.1	0, 100	0, 100	59.91 (28.80)	0.90
T3	185	32.7	0, 100	0, 100	60.17 (29.17)	0.91
T2–T1 change score	152	44.7	−100, 100	−100, 100	4.95 (27.10)	
T3–T2 change score	159	42.2	−100, 100	−100, 68	−0.85 (20.80)	
T3–T1 change score	154	44.0	−100, 100	−100, 100	4.96 (29.73)	
Trauma Symptoms						
T1	267	2.9	0, 100	0, 100	75.78 (19.79)	0.88
T2	230	16.4	0, 100	27, 100	81.80 (16.02)	0.84
T3	226	17.8	0, 100	0, 100	83.94 (17.33)	0.87
T2–T1 change score	223	18.9	−100, 100	−39, 61	5.79 (15.73)	
T3–T2 change score	215	21.8	−100, 100	−100, 39	1.94 (14.56)	
T3–T1 change score	219	20.4	−100, 100	−89, 61	7.80 (17.69)	
Positive Growth						
T1	264	4.0	0, 100	0, 100	38.27 (27.34)	0.83
T2	225	18.2	0, 100	0, 100	41.50 (28.00)	0.88
T3	223	18.9	0, 100	0, 100	44.52 (26.80)	0.89
T2–T1 change score	215	21.8	−100, 100	−100, 100	2.41 (30.36)	
T3–T2 change score	208	24.4	−100, 100	−67, 87	3.06 (24.16)	
T3–T1 change score	214	22.2	−100, 100	−100, 100	5.39 (28.62)	

**Table 4:** CARe Burn Scale—Adult Form correlations with comparison measures at each time point. Time 1 (T1: baseline, up to 4 weeks post-burn), Time 2 (T2: 3 months post-burn), and Time 3 (T3: 6 months post-burn).

CARe Burn Scale—Adult Form	Comparison Measure	r	95% Confidence Intervals
Wound/Scar Discomfort T1	EQ-5D-5L Pain Discomfort T1	−0.054 **	−0.62, −0.45
Wound/Scar Discomfort T2	EQ-5D-5L Pain Discomfort T2	−0.62 **	−0.70, −0.53
Wound/Scar Discomfort T3	EQ-5D-5L Pain Discomfort T3	−0.52 **	−0.61, −0.42
Wound/Scar Dissatisfaction T1	BSHS-B Body Image T1	0.64 **	0.56, 0.71
Wound/Scar Dissatisfaction T2	BSHS-B Body Image T2	0.71 **	0.64, 0.77
Wound/Scar Dissatisfaction T3	BSHS-B Body Image T3	0.67 **	0.59, 0.74
Physical Well-being T1	EQ-5D-5L T1	0.52 **	−0.60, −0.43
Physical Well-being T2	EQ-5D-5L T2	0.54 **	−0.63, −0.44
Physical Well-being T3	EQ-5D-5L T3	0.42 **	−0.52, −0.31
Social Situations T1	BSHS-B Body Image T1	0.49 **	0.39, 0.58
Social Situations T2	BSHS-B Body Image T2	0.58 **	0.49, 0.66
Social Situations T3	BSHS-B Body Image T3	0.62 **	0.53, 0.69
Self-Worth T1	MHI Depression T1	0.71 **	0.64, 0.78
Self-Worth T2	MHI Depression T2	0.67 **	0.59, 0.74
Self-Worth T3	MHI Depression T3	0.77 **	0.71, 0.82
Negative Mood T1	MHI Depression T1	0.72 **	0.66, 0.77
Negative Mood T2	MHI Depression T2	0.66 **	0.58, 0.73
Negative Mood T3	MHI Depression T3	0.66 **	0.58, 0.73
Work Life T1	BSHS-B Interpersonal Relationships T1	0.31 **	0.15, 0.46
Work Life T2	BSHS-B Interpersonal Relationships T2	0.44 **	0.30, 0.56
Work Life T3	BSHS-B Interpersonal Relationships T3	0.52 **	0.39, 0.63
Family Support T1	MSPSS Family T1	0.51 **	0.41, 0.59
Family Support T2	MSPSS Family T2	0.56 **	0.46, 0.64
Family Support T3	MSPSS Family T3	0.60 **	0.51, 0.68
Friend Support T1	MSPSS Friend T1	0.49 **	0.39, 0.58
Friend Support T2	MSPSS Friend T2	0.45 **	0.34, 0.55
Friend Support T3	MSPSS Friend T3	0.48 **	0.37, 0.57
Intimacy T1	BSHS-B Body Image T1	0.63 **	0.54, 0.71
Intimacy T2	BSHS-B Body Image T2	0.56 **	0.45, 0.65
Intimacy T3	BSHS-B Body Image T3	0.68 **	0.59, 0.75
Trauma Symptoms T1	PCL-C T1	−0.73 **	−0.78, −0.67
Trauma Symptoms T2	PCL-C T2	−0.64 **	−0.71, −0.56
Trauma Symptoms T3	PCL-C T3	−0.68 **	−0.71, −0.56
Positive Growth T1	PTGI-SF T1	0.41 **	0.30, 0.51
Positive Growth T2	PTGI-SF T2	0.42 **	0.31, 0.52
Positive Growth T3	PTGI-SF T3	0.53 **	0.43, 0.62

** Correlation is significant at the 0.01 level (2-tailed).

**Table 5:** CARe Burn Scale—Adult Form change score correlations with comparison measures at each time point. Time 1 (T1: baseline, up to 4 weeks post-burn), Time 2 (T2: 3 months post-burn), and Time 3 (T3: 6 months post-burn).

CARe Burn Scale—Adult Form Subscales Change Scores	Comparison Measure Change Scores	r	95% Confidence Intervals
Wound/Scar Discomfort (T2–T1)	EQ-5D-5L Pain Discomfort (T2-T1)	−0.31 **	−0.43, −0.18
Wound/Scar Discomfort (T3–T2)	EQ-5D-5L Pain Discomfort (T3-T2)	−0.24 **	−0.37, −0.11
Wound/Scar Discomfort (T3–T1)	EQ-5D-5L Pain Discomfort (T3-T1)	−0.32 **	−0.44, −0.19
Wound/Scar Dissatisfaction (T2–T1)	BSHS-B Body Image (T2-T1)	0.21 **	0.08, 0.33
Wound/Scar Dissatisfaction (T3–T2)	BSHS-B Body Image (T3-T2)	0.23 **	0.10, 0.35
Wound/Scar Dissatisfaction (T3–T1)	BSHS-B Body Image (T3-T1)	0.41 **	0.29, 0.52
Physical Well-being (T2–T1)	EQ-5D-5L Summary Index (T2-T1)	0.31 **	−0.42, −0.19
Physical Well-being (T3–T2)	EQ-5D-5L Summary Index (T3-T2)	0.25 **	−0.37, −0.12
Physical Well-being (T3–T1)	EQ-5D-5L Summary Index (T3-T1)	0.37 **	−0.48, −0.25
Social Situations (T2–T1)	BSHS-B (T2-T1)	0.17 *	0.03, 0.30
Social Situations (T3–T2)	BSHS-B (T3-T2)	0.16 *	0.02, 0.29
Social Situations (T3–T1)	BSHS-B (T3-T1)	0.18 *	0.04, 0.31
Self-Worth (T2–T1)	MHI Depression (T2-T1)	0.43 **	0.32, 0.53
Self-Worth (T3–T2)	MHI Depression (T3-T2)	0.38 **	0.26, 0.49
Self-Worth (T3–T1)	MHI Depression (T3-T1)	0.42 **	0.30, 0.52
Negative Mood (T2–T1)	MHI Depression (T2-T1)	0.34 **	0.22, 0.45
Negative Mood (T3–T2)	MHI Depression (T3-T2)	0.26 **	0.13, 0.38
Negative Mood (T3–T1)	MHI Depression (T3-T1)	0.41 **	0.29, 0.51
Work Life (T2–T1)	BSHS-B Interpersonal Relationships (T2-T1)	0.32 **	0.13, 0.49
Work Life (T3–T2)	BSHS-B Interpersonal Relationships (T3-T2)	0.11	−0.07, 0.28
Work Life (T3–T1)	BSHS-B Interpersonal Relationships (T3-T1)	0.24 *	0.04, 0.42
Family Support (T2–T1)	MSPSS Family (T2-T1)	0.38 **	0.26, 0.40
Family Support (T3–T2)	MSPSS Family (T3-T2)	0.20 **	0.06, 0.33
Family Support (T3–T1)	MSPSS Family (T3-T1)	0.35 **	0.22, 0.47
Friend Support (T2–T1)	MSPSS Friend (T2-T1)	0.22 *	0.09, 0.34
Friend Support (T3–T2)	MSPSS Friend (T3-T2)	0.12	−0.01, 0.25
Friend Support (T3–T1)	MSPSS Friend (T3-T1)	0.18 *	0.05, 0.31
Intimacy (T2–T1)	BSHS-B Body Image (T2-T1)	0.42 **	0.28, 0.54
Intimacy (T3–T2)	BSHS-B Body Image (T3-T2)	0.14	−0.02, 0.29
Intimacy (T3–T1)	BSHS-B Body Image (T3-T1)	0.42 **	0.28, 0.54
Trauma Symptoms (T2–T1)	PCL-C (T2-T1)	−0.33 **	−0.44, −0.21
Trauma Symptoms (T3–T2)	PCL-C (T3-T2)	−0.36 **	−0.47, 0.24
Trauma Symptoms (T3–T1)	PCL-C (T3-T1)	−0.48 **	−0.58, −0.37
Positive Growth (T2–T1)	PTGI-SF (T2-T1)	0.13	0.18, 0.43
Positive Growth (T3–T2)	PTGI-SF (T3-T2)	0.26 **	0.13, 0.38
Positive Growth (T3–T1)	PTGI-SF (T3-T1)	0.19 **	0.06, 0.32

* Correlation is significant at the 0.05 level (2-tailed). ** Correlation is significant at the 0.01 level (2-tailed).

**Table 6:** Final MID values for the CARe Burn Scale—Adult Form.

Subscale	Time	Final MID	% Smaller than MID (No Change)	% Greater or Equal to MID (Small Change)	% Greater or Equal to MID (Big Change)	Overall Accuracy	Overall Accuracy 95% CI *
Wound/Scar Discomfort	T2	5	33% (5/15)	57% (28/49)	88% (121/138)	76% (154/202)	70 to 82
	T3	5	60% (32/53)	52% (28/54)	51% (48/95)	53% (108/202)	47 to 60
Wound/Scar Dissatisfaction	T2	9	61% (45/74)	42% (39/93)	59% (30/51)	52% (114/218)	46 to 59
	T3	9	70% (79/113)	36% (24/67)	47% (16/34)	56% (119/214)	49 to 62
Physical Wellbeing	T2	12	38% (30/80)	53% (31/59)	76% (63/83)	56% (124/222)	49 to 62
	T3	12	81% (98/121)	37% (17/46)	47% (22/47)	64% (137/214)	57 to 70
Social Situations	T2	5	43% (41/96)	54% (32/59)	64% (32/50)	51% (105/205)	44 to 58
	T3	5	60% (72/120)	57% (29/51)	41% (16/39)	56% (117/210)	49 to 62
Self-Worth	T2	6	61% (71/117)	56% (37/66)	54% (20/37)	58% (128/220)	52 to 65
	T3	6	60% (83/139)	51% (25/49)	54% (13/24)	57% (121/212)	50 to 64
Negative Mood	T2	7	51% (57/111)	42% (30/72)	54% (22/41)	49% (109/224)	42 to 55
	T3	7	77% (98/128)	41% (24/59)	37% (10/27)	62% (132/214)	55 to 68
Work Life	T2	15	81% (46/57)	25% (6/24)	37% (7/19)	59% (59/100)	49 to 68
	T3	15	81% (72/89)	35% (6/17)	35% (6/17)	68% (84/123)	60 to 76
Family Support	T2	8	86% (144/167)	40% (6/15)	38% (11/29)	76% (161/211)	70 to 82
	T3	8	83% (141/169)	29% (4/14)	33% (4/12)	76% (149/195)	70 to 82
Friend Support	T2	6	63% (92/145)	42% (15/36)	51% (18/35)	58% (125/216)	51 to 64
	T3	6	66% (101/154)	66% (21/32)	58% (15/26)	65% (137/212)	58 to 71
Intimacy	T2	5	59% (49/83)	49% (18/37)	61% (19/31)	57% (86/151)	49 to 65
	T3	5	70% (78/112)	32% (9/28)	47% (9/19)	60% (96/159)	53 to 68
Trauma Symptoms	T2	4	59% (69/118)	59% (38/65)	43% (17/40)	56% (124/223)	49 to 62
	T3	4	71% (89/126)	40% (23/57)	50% (16/32)	60% (128/215)	53 to 66
Positive Growth	T2	9	69% (94/137)	51% (27/53)	52% (13/25)	62% (134/215)	56 to 69
	T3	9	64% (95/148)	48% (20/42)	44% (8/18)	59% (123/208)	52 to 66

* Binomial Proportion calculated by Wilson’s Method.

## Appendix A

MID calculation:

To calculate MIDs using anchor-based methods, the minimum threshold for the correlation between the anchor and the change score is ≥0.3 [20]. For each subscale of the CARe Burn Scale—Adult Form, a single-anchor transition question and the change scores were used to first calculate the MID values, using the T2 single item transition question and the change score between T2 and T1. These MID values were then examined using the T3 single item transition question and the change scores between T3 and T2. We calculated the change score, reversed the sign of the score for those reporting a poorer outcome, and used a derived self-reported anchor with categories of ‘no change’, ’small but important change’, and ‘large and important change’. Thereafter, the MID was the value of the change score for the outcome measure in the ‘small but important change’ group data such that it lies in the inter-quartile range and is close to the median. The specific value for the MID is that change score which jointly minimises the percentage of those reporting no change having outcome values greater than or equal to the MID while simultaneously minimising the percentage of those in the big change category having outcome values less than the MID.

For the distribution-based approach, the changes in comparison quality of life measures were used as an anchor. Absolute changes of less than 0.2 standard deviation were taken as ‘no change’; absolute changes of between 0.2 to 0.5 standard deviation were taken as a ‘small but important change’, and absolute change beyond 0.5 standard deviations wad taken as a ‘large and important change’. These thresholds are informed by the thresholds tentatively advanced by Cohen, where the absolute values of d under 0.2 SD are typically interpreted as representing a trivial or no change; between 0.2 and 0.5 SD as being a small effect, and 0.5 SD being the lower bound of a medium-sized effect. Thereafter, the same algorithmic process for identifying the MID in the anchor-based approach was used with the derived distribution anchor.

The same procedures for calculating the MIDs using the T1 and T2 data were additionally applied to the T2 and T3 data. Hence, distribution-based approaches were used to examine the consistency between the two approaches, and the long-term longitudinal data was used as an additional approach for consistency. In instances when there were differences between the MIDs developed via anchor and distributional methods, the anchor-based MIDs were retained as these focused directly on the phenomenon of interest, i.e., self-reported change in the domain, and specifically reflected the research question rather than a proxy measure of change used in the distribution-based approaches.

**Table A1:** CARe Burn Scale—Adult Form correlations with anchor questions at each time point. Time 1 (T1: baseline, up to 4 weeks post-burn), Time 2 (T2: 3 months post-burn), and Time 3 (T3: 6 months post-burn).

CARe Burn Scale—Adult Form Subscale	Anchor Question	r	95% Confidence Intervals
Wound/Scar Discomfort T1	Wound/Scar Discomfort T2	0.01	−0.13, 0.15
Wound/Scar Discomfort T2	Wound/Scar Discomfort T2	−0.39 **	−0.50, −0.27
Wound/Scar Discomfort T2	Wound/Scar Discomfort T3	−0.06	−0.20, −0.08
Wound/Scar Discomfort T3	Wound/Scar Discomfort T3	−0.08	−0.21, 0.05
Wound/Scar Dissatisfaction T1	Wound/Scar Dissatisfaction T2	−0.03	−0.16, 0.10
Wound/Scar Dissatisfaction T2	Wound/Scar Dissatisfaction T2	−0.23 **	−0.35, −0.10
Wound/Scar Dissatisfaction T2	Wound/Scar Dissatisfaction T3	0.02	−0.11, 0.15
Wound/Scar Dissatisfaction T3	Wound/Scar Dissatisfaction T3	−0.03	−0.16, 0.10
Physical Well-being T1	Physical Wellbeing T2	0.03	−0.10, 0.16
Physical Well-being T2	Physical Wellbeing T2	−0.16 *	−0.29, −0.03
Physical Well-being T2	Physical Wellbeing T3	0.11	−0.02, 0.24
Physical Well-being T3	Physical Wellbeing T3	−0.10	−0.23, 0.03
Social Situations T1	Social Situations T2	−0.06	−0.20, 0.08
Social Situations T2	Social Situations T2	−0.14 *	−0.27, −0.01
Social Situations T2	Social Situations T3	0.05	−0.09, 0.18
Social Situations T3	Social Situations T3	0.01	−0.12, 0.14
Self-Worth T1	Self-Worth T2	−0.10	−0.23, 0.03
Self-Worth T2	Self-Worth T2	−0.29 **	−0.16, −0.41
Self-Worth T2	Self-Worth T3	−0.07	−0.20, 0.07
Self-Worth T3	Self-Worth T3	−0.18 **	−0.30, −0.05
Negative Mood T1	Negative Mood T2	−0.03	−0.16, 0.10
Negative Mood T2	Negative Mood T2	−0.11	−0.24, 0.02
Negative Mood T2	Negative Mood T3	−0.01	−0.14, 0.12
Negative Mood T3	Negative Mood T3	−0.12	−0.25, 0.01
Work Life T1	Work Life T2	0.10	−0.09, 0.29
Work Life T2	Work Life T2	0.02	.14, 0.18
Work Life T2	Work Life T3	0.16	−0.01, 0.32
Work Life T3	Work Life T3	−0.04	−0.20, 0.12
Family Support T1	Family Support T2	0.12	−0.01, 0.25
Family Support T2	Family Support T2	−0.05	−0.18, 0.08
Family Support T2	Family Support T3	0.02	−0.12, 0.16
Family Support T3	Family Support T3	−0.05	−0.18, 0.09
Friend Support T1	Friend Support T2	0.04	−0.09, 0.17
Friend Support T2	Friend Support T2	−0.02	−0.15, 0.11
Friend Support T2	Friend Support T3	0.12	−0.01, 0.25
Friend Support T3	Friend Support T3	−0.05	−0.18, 0.08
Intimacy T1	Intimacy T2	−0.07	−0.22, 0.08
Intimacy T2	Intimacy T2	−0.24 **	−0.37, 0.10
Intimacy T2	Intimacy T3	0.03	−0.12, 0.18
Intimacy T3	Intimacy T3	−0.09	−0.23, 0.05
Trauma Symptoms T1	Trauma Symptoms T2	0.03	−0.10, 0.16
Trauma Symptoms T2	Trauma Symptoms T2	−0.08	−0.21, 0.05
Trauma Symptoms T2	Trauma Symptoms T3	0.07	−0.06, −0.18
Trauma Symptoms T3	Trauma Symptoms T3	−0.05	−0.18, 0.08
Positive Growth T1	Positive Growth T2	−0.27 **	−0.39, −0.14
Positive Growth T2	Positive Growth T2	−0.44 **	−0.54, −0.33
Positive Growth T2	Positive Growth T3	−0.30 **	−0.42, −0.17
Positive Growth T3	Positive Growth T3	−0.46 **	−0.56, −0.35

* Correlation is significant at the 0.05 level (2-tailed). ** Correlation is significant at the 0.01 level (2-tailed).

**Table A2:** CARe Burn Scale—Adult Form subscale change score correlations with anchor questions.

Change Score	Anchor Questions	r	95% Confidence Intervals
Wound/Scar Discomfort (T2–T1)	Wound/Scar Discomfort T2	−0.37 **	−0.48, −0.24
Wound/Scar Discomfort (T3–T2)	Wound/Scar Discomfort T3	−0.15 *	−0.28, −0.01
Physical Well-being (T2–T1)	Physical Well-being T2	−0.17 **	−0.30, −0.04
Physical Well-being (T3–T2)	Physical Well-being T3	−0.20 **	−0.33, −0.07
Social Situations (T2–T1)	Social Situations T2	−0.13	−0.26, 0.01
Social Situations (T3–T2)	Social Situations T3	−0.12	−0.25, 0.02
Friend Support (T2–T1)	Friend Support T2	−0.07	−0.20, 0.06
Friend Support (T3–T2)	Friend Support T3	−0.23 **	−0.35, −0.10
Work Life (T2–T1)	Work Life T2	−0.02	−0.22, 0.18
Work Life (T3–T2)	Work Life T3	−0.20 *	−0.36, −0.02
Family Support (T2–T1)	Family Support T2	−0.18 **	−0.31, −0.05
Family Support (T3–T2)	Family Support T3	−0.04	−0.18, 0.10
Self Worth (T2–T1)	Self-worth T2	−0.23 **	−0.35, −0.10
Self Worth (T3–T2)	Self-worth T3	−0.19 **	−0.32, −0.06
Wound/Scar Dissatisfaction (T2–T1)	Wound/Scar Dissatisfaction T2	−0.16 *	−0.29, −0.03
Wound/Scar Dissatisfaction (T3–T2)	Wound/Scar Dissatisfaction T3	−0.07	−0.20, 0.06
Intimacy (T2–T1)	Intimacy T2	−0.20 *	−0.35, −0.04
Intimacy (T3–T2)	Intimacy T3	−0.04	−0.19, 0.12
Trauma Symptoms (T2–T1)	Trauma Symptoms T2	−0.08	−0.21, 0.05
Trauma Symptoms (T3–T2)	Trauma Symptoms T3	−0.15 *	−0.28, −0.02
Negative Mood (T2–T1)	Negative Mood T2	−0.07	−0.20, 0.06
Negative Mood (T3–T2)	Negative Mood T3	−0.13	−0.26, 0.00
Positive Growth (T2–T1)	Positive Growth T2	−0.15 *	−0.28, −0.02
Positive Growth (T3–T2)	Positive Growth T3	−0.13	−0.26, 0.01

* Correlation is significant at the 0.05 level (2-tailed). ** Correlation is significant at the 0.01 level (2-tailed).

**Table A3:** CARe Burn Scale—Adult Form—anchor-based MID results.

Subscale	Time	MID	% Smaller than MID (No Change)	% Greater or Equal to MID (Small Change)	% Greater or Equal to MID (Big Change)	Overall Accuracy	Overall Accuracy 95% CI
Wound/Scar Discomfort	T2	5	33% (5/15)	57% (28/49)	88% (121/138)	76% (154/202)	70 to 82
	T3	5	60% (32/53)	52% (28/54)	51% (48/95)	53% (108/202)	47 to 60
Wound/Scar Dissatisfaction	T2	9	61% (45/74)	42% (39/93)	59% (30/51)	52% (114/218)	46 to 59
	T3	9	70% (79/113)	36% (24/67)	47% (16/34)	56% (119/214)	49 to 62
Physical Well-being	T2	12	38% (30/80)	53% (31/59)	76% (63/83)	56% (124/222)	49 to 62
	T3	12	81% (98/121)	37% (17/46)	47% 22/47)	64% (137/214)	57 to 70
Social Situations	T2	5	43% (41/96)	54% (32/59)	64% (32/50)	51% (105/205)	44 to 58
	T3	5	60% (72/120)	57% (29/51)	41% (16/39)	56% (117/210)	49 to 62
Self-Worth	T2	6	61% (71/117)	56% 37/66)	54% (20/37)	58% (128/220)	52 to 65
	T3	6	60% (83/139)	51% (25/49)	54% (13/24)	57% (121/212)	50 to 64
Negative Mood	T2	7	51% (57/111)	42% (30/72)	54% (22/41)	49% (109/224)	42 to 55
	T3	7	77% (98/128)	41% (24/59)	37% (10/27)	62% (132/214)	55 to 68
Work Life	T2	15	81% (46/57)	25% (6/24)	37% (7/19)	59% (59/100)	49 to 68
	T3	15	81% (72/89)	35% (6/17)	35% (6/17)	68% (84/123)	60 to 76
Family Support	T2	8	86% (144/167)	40% (6/15)	38% (11/29)	76% (161/211)	70 to 82
	T3	8	83% (141/169)	29% (4/14)	33% (4/12)	76% (149/195)	70 to 82
Friend Support	T2	6	63% (92/145)	42% (15/36)	51% (18/35)	58% (125/216)	51 to 64
	T3	6	66% (101/154)	66% (21/32)	58% (15/26)	65% (137/212)	58 to 71
Intimacy	T2	5	59% (49/83)	49% (18/37)	61% (19/31)	57% (86/151)	49 to 65
	T3	5	70% (78/112)	32% (9/28)	47% (9/19)	60% (96/159)	53 to 68
Trauma Symptoms	T2	4	59% (69/118)	59% (38/65)	43% (17/40)	56% (124/223)	49 to 62
	T3	4	71% (89/126)	40% (23/57)	50% (16/32)	60% (128/215)	53 to 66
Positive Growth	T2	9	69% (94/137)	51% (27/53)	52% (13/25)	62% (134/215)	56 to 69
	T3	9	64% (95/148)	48% 20/42)	44% (8/18)	59% (123/208)	52 to 66

**Table A4:** CARe Burn Scale—Adult Form—distribution-based MID results.

Subscale	Time	MID	% Smaller than MID (No Change)	% Greater or Equal to MID (Small Change)	% Greater or Equal to MID (Big Change)	Overall Accuracy	Overall Accuracy 95% CI
Wound/Scar Discomfort	T2	5	35% (20/58)	79% (80/101)	91% (38/42)	69% (138/201)	62 to 75
	T3	5	62% (89/144)	61% (30/49)	67% (6/9)	62% (125/202)	55 to 68
Wound/Scar Dissatisfaction	T2	9	55% (44/80)	38% (15/40)	50% (47/95)	49% (106/215)	43 to 56
	T3	9	73% (66/90)	45% (18/40)	43% (36/83)	56% (120/213)	50 to 63
Physical Wellbeing	T2	12	56% (14/25)	49% (24/49)	75% (110/147)	67% (148/221)	61 to 73
	T3	12	79% (72/91)	39% (27/70)	43% (23/53)	57% (122/214)	50 to 63
Social Situations	T2	5	51% (37/72)	50% (20/40)	55% (51/92)	53% (108/204)	46 to 60
	T3	5	71% (63/89)	22% (8/37)	48% (40/83)	53% (111/209)	46 to 60
Self-Worth	T2	6	70% (44/63)	48% (27/56)	69% (69/100)	64% (140/219)	57 to 70
	T3	6	63% (50/80)	44% (23/52)	63% (50/79)	58% (123/211)	52 to 65
Negative Mood	T2	6	57% (37/65)	35% (20/57)	60% (61/101)	53% (118/223)	46 to 59
	T3	6	82% (66/81)	36% (19/53)	44% 35/79)	56% (120/213)	50 to 63
Work Life	T2	12	73% (24/33)	26% (9/35)	31% (10/32)	43% (43/100)	34 to 53
	T3	15	86% (50/58)	30% (11/37)	43% (12/28)	59% (73/123)	51 to 68
Family Support	T2	7	78% (66/85)	37% (18/49)	51% (39/76)	59% (123/210)	52 to 65
	T3	7	83% (76/92)	38% (15/40)	32% (20/63)	57% (111/195)	50 to 64
Friend Support	T2	6	65% (54/83)	44% (30/68)	57% (37/65)	56% (121/216)	49 to 62
	T3	6	58% (55/95)	40% (25/63)	41% (22/54)	48% (102/212)	41 to 59
Intimacy	T2	5	72% (36/50)	57% (17/30)	60% (42/70)	63% (95/150)	55 to 71
	T3	5	65% (43/66)	36% (11/31)	46% (28/61)	52% (82/158)	44 to 60
Trauma Symptoms	T2	3	70% (52/74)	31% (17/55)	18% (16/87)	39% (85/216)	33 to 46
	T3	3	71% (61/86)	26% (13/51)	18% (13/74)	41% (87/211)	35 to 48
Positive Growth	T2	9	60% (35/58)	35% (12/34)	42% (50/118)	46% (97/210)	40 to 53
	T3	9	69% (42/61)	44% (24/55)	48% (44/91)	53% (110/207)	46 to 60
